# Dry versus wet dormancy: suspended lives of *Bacillus subtilis* versus *Saccharomyces cerevisiae* spores

**DOI:** 10.1128/msystems.00494-25

**Published:** 2026-05-15

**Authors:** Hyun Youk

**Affiliations:** 1 Department of Physics, University of Illinois Urbana-Champaign 14589, Urbana, Illinois, USA; 2 NSF Science and Technology Center for Quantitative Cell Biology, University of Illinois Urbana-Champaign 14589, Urbana, Illinois, USA; 3 Grainger College of Engineering, University of Illinois Urbana-Champaign 14589, Urbana, Illinois, USA; CNRS Delegation Alpes, Villeurbanne, Rhône-Alpes, France

**Keywords:** dormancy, spores, *Bacillus subtilis*, *Saccharomyces cerevisiae*, mathematical modeling, dynamical systems, single-cell studies

## Abstract

Dormant microbial spores provide one of the clearest and most extreme examples of how cells can pause life for extended periods and then reliably restart it. Although bacterial and fungal spores are often grouped under “dormancy,” the physical strategies by which they suspend and resume life differ fundamentally. Here, I compare two canonical systems—*Bacillus subtilis* endospores and *Saccharomyces cerevisiae* ascospores—using a dynamical-systems framework from a physicist’s perspective. I propose that dormancy is not simply “low metabolism,” but a dynamical reconfiguration that decouples local molecular clocks from a global biological clock while preserving an intrinsic capacity to resume sustained nonequilibrium dynamics, which I refer to as nonequilibrium capacity. Specifically, in *B. subtilis* spores, “dry dormancy” is enforced by immobilization: dehydration and material constraints suppress appreciable molecular diffusion and reaction fluxes, arresting global biological time by suppressing local molecular clocks. In *S. cerevisiae* spores, “wet dormancy” appears to be achieved by throttling: spores remain hydrated, retain molecular mobility, and support some slow irreversible processes such as gene expression, yet global biological time remains arrested because, as I propose, local activity fails to propagate into sustained organism-level progression (e.g., growth and division). Together, these comparisons place dry and wet dormancy as distinct regions of a physical design space defined by hydration, molecular mobility, energetic flux, and cross-scale coupling between local activity and global progression, and motivate quantitative models of dormancy and revival dynamics.

## INTRODUCTION

## SPORES AS TESTBEDS AT THE BOUNDARY OF LIVING AND NONLIVING

Spores have long fascinated microbiologists, physicists, and philosophers because they blur a boundary that is deceptively ingrained in most people’s minds as being crisp. A dormant spore can appear lifeless, showing no growth and little to no detectable metabolic activity. Yet, upon nutrient exposure, dormant spores can restart the dynamical, self-organizing hallmarks of life, including gene expression, energy transduction, and self-replication. This ability makes spores unusually powerful testbeds for developing a physical theory of living matter that explains how a system can persist for long periods in a deeply slowed state and then, when the influx of matter and energy resumes, transition back into a regime of sustained nonequilibrium activity (1).

In a thermodynamic sense, spore germination marks the re-igniting of life: the cell restarts maintenance of its local order by consuming free energy and exporting entropy to the environment (2). In other words, spore awakening marks a return to the canonical living mode: continuous dissipation that supports structured dynamics (3). In this paper, I motivate a more general framing of dormancy: not simply “low metabolism,” but a reconfiguration of internal physical state variables that preserves a cell’s latent capacity to produce and sustain nonequilibrium dynamics when conditions allow—a property I recently articulated as a cell’s nonequilibrium capacity (NEC) (1).

Not all microbes form spores under starvation, and among those that do, *Bacillus subtilis* and *Saccharomyces cerevisiae* spores are arguably the most intensively studied systems (4, 5). *B. subtilis* exemplifies bacterial (prokaryotic) endospores, whereas *S. cerevisiae* exemplifies fungal (eukaryotic) ascospores. Their biological differences are obvious: sporulation programs, germination processes, and spore wall architectures are substantially different. Their physical (material) differences are also apparent at the level of spore layers and barriers. Yet an additional difference, one that is less visually obvious but conceptually important, is the degree of internal dynamical activity during dormancy, which has been rarely studied in the framework of dynamical systems and is the focus of this paper.

It is worth clarifying at the outset the sense in which I use the term “dormancy” in this paper. The term is used broadly in the microbiology literature to cover many states in which cells are not actively growing, including antibiotic-induced persistence, stationary-phase arrest, quiescence, and the survival of microbes in extreme environments such as deep-sea sediments or desiccated soils. All of these states raise important biological questions, and I do not intend to exclude them from the broader conversation about dormancy. In this paper, however, I use the term in a narrower and more specific sense: the deeply arrested state produced by sporulation. Sporulation is a genetically encoded developmental program, involving the coordinated action of many genes and executing over several hours. It morphologically transforms the cell, actively engineers its internal physical state, and packages molecular resources in forms suited for long-term stability and reliable revival. The resulting spore is therefore not simply a cell that has stopped growing under adverse conditions, but a cell that has been programmatically remodeled for suspended animation. Both *B. subtilis* endospores and *S. cerevisiae* ascospores are produced through such programs, which is what makes them well-defined and quantitatively tractable model systems for developing a physical theory of suspended life. In this paper, I do not treat antibiotic persistence, stationary-phase arrest, or near-zero-growth states as dormancy in the same sense as spores, because they do not involve the same developmental remodeling and self-engineered physical state. I note in a later section where such states may fit within the broader physical design space that I propose.

A related conceptual question concerns the boundary between very slow growth and complete developmental arrest. A recent study of slowly growing yeast demonstrates that cell cycle progression cannot be sustained below a critical rate: reactive oxygen species (ROS) accumulate faster than gene expression can reduce them at frigid temperatures, such that cells with sufficiently high ROS concentrations fail to complete the cell cycle and die, imposing a lower bound on the viable cell cycle progression rate (6). Supported by quantitative experiments and a thermodynamics-based model of gene expression, this work showed that a continuous transition from finite growth rates (growth rate [μ] > 0) to complete arrest (μ = 0) need not exist—and may in fact be dynamically inaccessible, with cells unable to survive with arbitrarily low growth rates. More generally, it suggests that transitions from growing to nongrowing states need not arise from a gradual slowing of the same underlying dynamics, but can instead involve a reorganization of how intracellular molecular processes are coupled to global cellular progression. Sporulation-derived dormancy is one well-defined example of such a transition.

As useful shorthand, we can distinguish “dry dormancy” and “wet dormancy,” terms I introduce here. I use these terms as physical shorthand for extremes along a spectrum of physical state variables, primarily hydration and molecular mobility, rather than as taxonomic categories, exhaustive classifications, or positions on a growth-rate continuum. In *B. subtilis*, the spore core is “dry” in the sense that water content and activity are greatly reduced to extremely low levels, and the cytoplasm becomes strongly immobilized (4, 7–10). Consequently, most molecular diffusion and chemical reactions in the core are thought to be severely suppressed to levels that are negligible on biologically relevant timescales, even if not strictly zero. Consistent with this view, ATP is widely believed to be absent or chemically inaccessible in dormant *B. subtilis* spores, although direct single-molecule ATP measurements in single spores remain challenging. Importantly, “dry” does not mean a literal absence of molecular motion: at finite temperature (above absolute zero), some residual fluctuations necessarily persist. Residual hydration remains in dormant spores, and rare, strongly constrained molecular fluctuations and diffusion would still occur (7). Rather than eliminating dynamics entirely, the defining feature of dry dormancy is that residual molecular motion, including intramolecular conformational fluctuations and highly constrained intermolecular diffusion, remains far below the level needed for macromolecular transport and is effectively decoupled from productive chemical pathways. In yeast, by contrast, spores are “wet”: the cytoplasm remains hydrated and viscoelastic, with measurable molecular mobility (11). A recent study showed that dormant yeast spores retain detectable protein mobility, allowing components of gene expression machinery to move and function, albeit slowly, producing measurable transcripts and proteins. Thus, wet dormancy is not a static arrested state but a regime of reduced yet finite dynamics (11). This distinction raises questions that are difficult to formulate if dormancy is treated as a single, uniform physical state across microbes.

Several underexplored questions follow naturally from placing dry and wet spores side by side. What is it about sporulation, as a dedicated developmental program, that enables spore-forming cells to suspend life for extended periods and reliably restart it, in ways that cells not executing an equivalent program generally cannot achieve? What dynamical features distinguish wet dormancy from dry dormancy beyond the molecular parts list? Are there organizing principles, at the level of dynamical-systems state variables and phase-space structure, that allow life to be suspended? In this article, I use *B. subtilis* and yeast spores as anchor points to discuss these questions. I focus especially on quantitative puzzles that emerge in dry and wet dormancies. Our thesis is that a deep understanding of dormancy would arise from studying it not only biochemically but also as a problem in dynamical systems: how a system with heterogeneous initial configurations—exhibited by spore-to-spore variability within isogenic populations (11–15)—and slow internal dynamics can transition into a robust region of state space corresponding to the resumption of active life.

## DORMANCY AS A DYNAMICAL-SYSTEMS PROBLEM: STOPPING GLOBAL TIME WITH LOCAL CLOCKS

A dynamical system is a rule that maps a system’s current state to its future state (16, 17). In biological contexts, this language is often used to describe gene-regulatory circuits, metabolic networks, and cell fate decisions, where the central goal is not only to compute individual trajectories but also to understand global structure, such as attractors and their basins, metastable states, and transitions between them (18, 19).

Dormant spores present a particularly extreme and underexplored case. A dormant spore is a system that appears frozen at the macroscopic level, showing no growth or developmental progression, yet remains capable of reliably restarting life when conditions permit. From a dynamical-systems perspective, this raises a fundamental question: what does it mean for “time” to stop in a biological system?

Here, I propose to frame dormancy in terms of local molecular clocks and a global biological clock. Local clocks refer to irreversible microscopic processes. These include transcription, translation, and chemical reactions. Local clocks define time at the level of individual molecules or reaction events. The global biological clock, by contrast, refers to the system-level progression associated with life—growth, division, developmental advance, and sustained nonequilibrium organization—rather than to chronological time *per se*. In this framework, actively growing cells correspond to a regime in which these clocks are tightly coupled, such that local irreversible processes propagate upward through feedback loops to drive global progression. Dormancy, by contrast, corresponds to a decoupling of these clocks.

**Fig 1:**
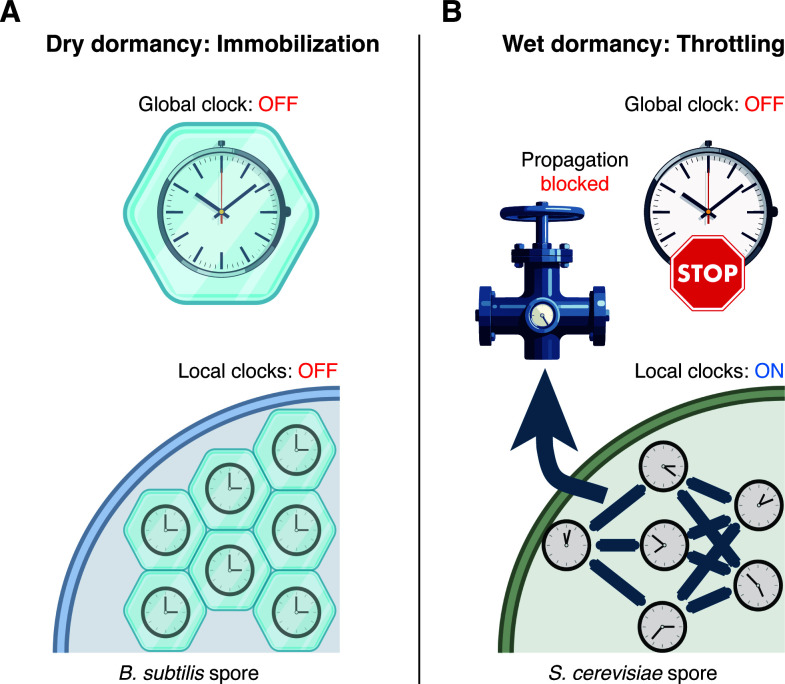
Stopping global biological time by immobilization or throttling. Two distinct physical strategies by which spores suspend life while preserving the capacity for revival. (**A**) Dry dormancy (immobilization). In systems such as *Bacillus subtilis* endospores, dehydration and physicochemical constraints drive the cytoplasm into a low-mobility, glass-like state. Molecular diffusion and reaction fluxes are strongly suppressed, rendering local molecular clocks (e.g., transcription, translation, and other irreversible microscopic processes) effectively nonproductive. Consequently, global biological time—defined as system-level progression associated with growth, division, and sustained self-organization—is arrested largely because local clocks themselves are suppressed. (**B**) Wet dormancy (throttling). In systems such as *Saccharomyces cerevisiae* ascospores, the cytoplasm remains hydrated and dynamically reorganizable, allowing local molecular clocks to continue running, albeit slowly and intermittently. However, one or more global constraints (e.g., energetic flux, physicochemical state, or coupling among local clocks) throttle the propagation of local activity into system-level feedback loops. Consequently, global biological time remains arrested despite ongoing microscopic dynamics.

In dry dormancy, exemplified by *B. subtilis* endospores, this decoupling is achieved in the most direct way. Dormancy is enforced by driving the system into a physical state in which molecular diffusion, chemical reactions, and energy transduction are so strongly suppressed that even at the level of individual molecules, productive dynamics are effectively halted at the scales relevant for macromolecular transport and network-level flux (4, 7, 8, 10). In this regime, global biological time stops because local molecular clocks are largely arrested or rendered nonproductive (Fig. 1A). The system occupies a region of state space in which motion itself is severely constrained and irreversible processes are minimized.

Wet dormancy, exemplified by *S. cerevisiae* spores (5), poses a deep and nontrivial conceptual challenge that is yet to be resolved. Here, the cytoplasm remains hydrated and viscoelastic, molecular mobility persists (including diffusion at the single-molecule level), and irreversible processes, including gene expression (11), can still occur. At the level of local molecular clocks, time continues to flow. Yet at the level of the organism, biological time appears stopped: spores do not grow, divide, or advance developmentally. From a dynamical-systems viewpoint, wet dormancy therefore raises a more subtle question: how can macroscopic time be arrested when microscopic clocks continue to tick?

To address this distinction, I propose here the concept of throttling of dynamics, a mode of dormancy that is distinct from immobilization and, to our knowledge, has not been explicitly articulated in the context of microbial spores. By throttling, I mean a regime in which molecular components and reaction networks remain physically present and, in some cases, dynamically active, but are held below the thresholds required for sustained, self-amplifying nonequilibrium behavior. Operationally, throttling implies that a small number of state variables (e.g., energetic flux, physicochemical state) set a ceiling on system-wide propagation of local activity (Fig. 1B). Importantly, throttling is not equivalent to uniform downscaling of all processes. Instead, it is a selective failure of propagation from local activity to global progression. In practice, such global variables may correspond to integrated energetic fluxes, physicochemical state variables, or network-level coupling strengths, rather than to individual molecular activities. This hypothesis makes testable predictions. For example, it predicts that awakening is controlled by threshold crossings in a small number of global variables rather than by a uniform resumption of all processes. Throttling does not eliminate motion or irreversibility; instead, it prevents local dynamics from propagating into global progression. In state-space terms, trajectories remain confined to a region that does not readily access basins corresponding to growth.

This distinction clarifies why dry and wet dormancy represent fundamentally different solutions to the same problem. In dry dormancy, escape from the dormant region of state space is prevented by suppressing motion in state space itself. In wet dormancy, escape is prevented by decoupling ongoing microscopic dynamics from the feedback loops that normally drive growth, division, and developmental time forward. In the latter case, dormancy corresponds not to a static point in state space, but to a region in which trajectories fluctuate or circulate without reaching a region of state space corresponding to active life.

A dynamical-systems framing also naturally accommodates heterogeneity. Even within genetically identical populations, spores differ in molecular composition and internal organization at the time dormancy is established, corresponding to different initial conditions within the dormant region of state space (11–14, 20). These differences can shape how and when individual spores eventually escape dormancy, without requiring distinct genetic programs or predetermined fates. This perspective is consistent with single-cell studies of dormancy and revival across microbial systems (11–14). Viewed through this lens, nutrient availability does not act as a simple on–off switch. Instead, nutrient repletion modifies constraints and reshapes the landscape of allowed dynamics. In dry dormancy, this reshaping enables motion itself to resume. In wet dormancy, it allows existing motion to become productive by restoring coupling between local processes and global feedback loops.

In the sections below, I apply this framework to two canonical spore-forming systems. *B. subtilis* endospores illustrate dormancy through enforced immobilization, where both local and global clocks are largely stopped. *S. cerevisiae* spores illustrate dormancy through throttling, where local clocks continue to run while global biological time remains arrested. Comparing these two cases highlights not only distinct molecular mechanisms, but also fundamentally different dynamical strategies for suspending life.

## DRY DORMANCY AS ENFORCED IMMOBILIZATION IN *BACILLUS SUBTILIS* SPORES

The mature endospore of *B. subtilis* is often described as “metabolically inactive,” but that phrasing masks an important concept. What makes the *B. subtilis* spore such a powerful model of dormancy is that it is not merely a cell that has turned metabolism off; it is a cell that has actively re-engineered its interior physical state so that most chemistry becomes kinetically inaccessible (4, 7, 10, 21–23). *B. subtilis* enters dormancy through enforced immobilization: it drives its core toward a low-mobility, low-water-activity regime that suppresses molecular mobility, conformational fluctuations, and therefore reaction fluxes (4, 8). Dry dormancy arrests the global biological clock largely by arresting the local molecular clocks. It does so not by selectively throttling a few pathways, but by pushing much of intracellular dynamics far below the threshold of productive chemistry. This makes *B. subtilis* spores a canonical case in which dormancy is best understood as a physical problem: how to build a cytoplasm that is functionally near-static while still preserving the capacity to restart.

A central physical aspect of *B. subtilis* spores is the dehydrated spore core. Classic measurements, reviewed extensively in the spore literature, show that the core water content is dramatically reduced relative to vegetative cells and that the remaining water is not simply “less,” but is constrained, exhibiting properties consistent with a highly crowded, glass-like cytoplasm (4, 7, 10). Specifically, the dormant *B. subtilis* spore core water content is approximately 35% of wet weight, rising to ~45% after Ca-DPA release (stage I germination) and to ~80% after cortex hydrolysis (stage II germination). This is more than a twofold difference between the dormant and fully germinated core (4). Note, however, an important caveat: NMR relaxation measurements indicate that water mobility in the spore core is higher than a strictly glass-like model would predict (7), suggesting that dormancy enforcement may involve dehydration-induced conformational constraints on key enzymes as much as glass-like quenching of water diffusion *per se*. The central physical point, which is that molecular diffusion and productive chemistry are strongly suppressed in dormant *B. subtilis* spores, nevertheless stands. Importantly, this core state is actively constructed during sporulation through a coordinated set of structural and chemical changes. One major contributor is the accumulation of Ca–dipicolinic acid (Ca-DPA) to extraordinarily high levels, often cited as a substantial fraction of spore dry weight, which lowers water activity and is intimately tied to core dehydration, heat resistance, and long-term stability (9). The dehydrated core is further stabilized by small acid-soluble spore proteins (SASPs) that saturate the chromosome to alter its conformation (24, 25), thereby protecting it from multiple forms of damage. Together, Ca-DPA accumulation and SASP-mediated DNA packaging are widely viewed as the core design elements that make the *B. subtilis* spore interior chemically “quiet,” which is more than being just metabolically downregulated, as in stationary-phase bacteria (4).

A second pillar of enforced immobilization is mechanical constraint. The *B. subtilis* spore is not a naked dehydrated sphere; its low-mobility core is maintained by intricate architecture surrounding it. The specialized cortex peptidoglycan and multilayer protein coat are often introduced as “protective barriers,” but in physical terms the cortex also functions as a mechanical device that helps maintain the core in a compressed, low-water state until germination (26, 27). This is why one can view germination of *B. subtilis* spores as a controlled phase transition: the spore must reverse the very constraints that maintain immobilization. Nutrients sensed by germinant receptors (i.e., without requiring ongoing ATP expenditure) initiate a cascade that includes Ca-DPA release, core rehydration, and cortex hydrolysis (4, 22, 28). In this view, germination does not require active, energy-consuming sensing programs. Instead, ligand binding to pre-existing receptors triggers conformational and biophysical changes that unlock ion fluxes and hydration dynamics (4, 28–31). Only after these early physical transitions restore molecular mobility can intracellular energy transduction and biosynthetic activity resume, and germination proceed. Physically, germination reopens local clocks (ion fluxes, hydration, molecular mobility), which then recouple to global clock progression.

If immobilization is the goal, then one would expect directly measurable signatures: protein mobility should be minimal in dormant *B. subtilis* spores and should increase sharply upon germination. Elegant imaging-based experiments have historically supported exactly this expectation, showing that a soluble GFP fusion protein is effectively immobile in dormant *B. subtilis* spores (diffusion coefficient ≤ 10⁻¹² cm²/s) but freely mobile in germinated spores (diffusion coefficient > 10⁻⁸ cm²/s) (8). This difference of at least four orders of magnitude provides direct evidence that the dormant *B. subtilis* spore’s core occupies a physical state that fundamentally differs from the cytoplasm after germination. These results are important because they cast dormancy as a measurable physical state variable, namely mobility, rather than inferred metabolic inactivity. They also establish a clear contrast with dormant yeast spores, whose interior remains hydrated and measurably dynamic (11).

A third, often underappreciated, aspect of dry dormancy is that the spore does not merely avoid chemical reactions. Instead, sporulation actively preserves the molecular machinery required to restart reactions without rebuilding everything from scratch. This raises a deep problem: if translation must be suppressed to conserve resources, how does a cell avoid suppressing it in a way that irreversibly destroys its own capacity to restart? Several layered answers are suggested by mechanistic studies in *B. subtilis* cells. Some have been established directly in the developing forespore and spore; others come from quiescent and stationary-phase cells and remain to be established in spores specifically. Jonathan Dworkin and Caroline Harwood have emphasized that a central challenge of quiescence is maintaining translational competence: keeping ribosomes structurally intact and poised for the return to growth (22). Dworkin’s work showed that a ribosome dimerization protein, hibernation-promoting factor (HPF), acts as a structural safeguard in stationary-phase cells: HPF-mediated ribosome dimerization preserves essential small-subunit proteins, notably S2 and S3, and mutants defective in HPF function show compromised ribosome integrity and impaired recovery (32). Whether analogous ribosome protection mechanisms operate in *B. subtilis* spores specifically remains to be established, but the principle—that translational competence must be actively protected, not merely passively conserved—may be relevant to understanding spore restart competence.

Complementing ribosome-level protection, Dworkin and colleagues have elucidated clear and elegant mechanisms for downregulating both translation initiation and elongation that are relevant to dormancy entry. First, phosphorylation of elongation factor Tu (EF-Tu) by the YabT kinase inhibits elongation by impairing EF-Tu’s GTPase cycle; this mechanism operates specifically in the developing forespore and mature spore (21). Second, in quiescent and stationary-phase cells, the alarmones (p)ppGpp directly regulate translation initiation during entry into quiescence by targeting initiation factor IF2 (20, 33); whether an analogous mechanism operates during sporulation and in the resulting spores remains an open question. Together, these findings underscore a conceptual point: entry into nongrowth states in *B. subtilis* involves not a blunt shutdown of ATP production, but multiple, layered kinetic constraints that reversibly suppress chemical fluxes through targeted interventions at specific molecular steps.

The stringent response and its nucleotide signals (GTP, (p)ppGpp) also provide a natural bridge between the biological framing of sporulation and the physicist’s framing of dormancy I am advocating here. In quiescent and nutrient-limited contexts, shifts in GTP and (p)ppGpp reshape transcriptional programs through regulators such as CodY, making nucleotide economy a central consideration for entry into nongrowth states (22). Conceptually, in a dynamical-systems framework, (p)ppGpp is powerful precisely because it couples an environmental condition to a global reconfiguration of the system’s dynamical possibilities: a small molecule that may push a cell toward a low-flux region of state space.

A major reason *B. subtilis* spores have become central to dormancy research—aside from their historical importance as the subject of Robert Koch’s and Ferdinand Cohn’s studies (along with *B. anthracis*)—is that they allow one to cleanly separate two questions that are often conflated: how does a cell stop, and how does a cell preserve the ability to restart? Studies from the groups of Setlow, Dworkin, Bischofs, and colleagues have been influential and important precisely because they took the second question seriously and pursued it mechanistically. This is also where phenotypic heterogeneity emerges as part of nature’s design for sporulation. Work from Ilka Bischofs’ group provides a particularly incisive example: they demonstrated that *B. subtilis* exhibits a spore quantity–quality tradeoff that links dormancy entry to dormancy exit through phenotypic memory, meaning that conditions and decisions made during sporulation shape the kinetics and success of germination (12, 13). This result is conceptually powerful because it shows that a spore’s “initial condition” in physical state space is not determined solely by genetics, but is shaped by the cell’s history in ways that measurably influence its revival later.

Relatedly, Dworkin’s own work has helped articulate how heterogeneity can act as a route out of dormancy rather than merely a byproduct of it. A clear statement of this idea is that phenotypic diversity can function as a population-level strategy for dormancy exit, enabling different individuals to explore distinct revival trajectories and timings (14, 15, 20). If the spore core is physically immobilized, then reactivation barriers will not be crossed uniformly. Instead, escape from dormancy proceeds through discrete molecular events whose timing naturally varies across individuals. Such events could include receptor activation, ion fluxes, or cortex hydrolysis. From this perspective, the long-standing phenomenon of “superdormant” spores (34) is not necessarily a distinct subclass, but a natural consequence of a system in which deep immobilization generates a distribution of exit barriers (12, 13).

Perhaps the most convincing way to connect this conceptual picture to physiology is to follow the return of metabolism at single-spore resolution. Here, Dworkin and colleagues’ recent contributions stand out. Single-spore bioluminescence measurements have made it possible to observe metabolic dynamics during germination and outgrowth directly, revealing striking heterogeneity in timing and intensity even among genetically identical spores (14). These experiments matter because they test a central physical claim: if dormancy is enforced immobilization, then revival should not be a smooth ramp but a sequence of transitions whose statistics reflect underlying physical constraints and molecular bottlenecks.

Taken together, these results support a concise summary of dry dormancy in *B. subtilis* as a strategy built from three coupled design principles. First, reaction fluxes are suppressed by pushing the core into a low-water, low-mobility, glass-like regime supported by Ca-DPA accumulation, SASP-mediated DNA protection, and mechanically enforced architecture (4, 8, 9, 27). Second, restart competence is preserved by stabilizing and protecting key macromolecular machines, especially the translation apparatus. Phosphorylation of elongation factor Tu (EF-Tu) inhibits translation elongation in the developing forespore (21). Additional mechanisms identified in quiescent *B. subtilis* cells—including HPF-mediated ribosome dimerization and targeted inhibition of translation initiation by (p)ppGpp (20, 32, 33)—may also be relevant, though their specific roles in spores remain to be established. Third, phenotypic heterogeneity is exploited, coupling entry conditions to exit outcomes and producing history-dependent distributions of germination behavior.

*B. subtilis* spores remain the canonical dry dormancy system because they offer an unusually explicit demonstration that dormancy is not a point on a spectrum of metabolic activity, but a constructed physical state with measurable material properties and a built-in route back to life. In the next section, this framing will sharpen the comparison to wet dormancy in *S. cerevisiae* spores, where internal mobility and hydration are preserved to a far greater extent even while development is arrested, suggesting that the deep question is which region of physical state space is inhabited by different dormant states, whether dry or wet. Together, the studies mentioned in this section have transformed how dormancy is studied in *B. subtilis*, shifting the focus of dormancy from static endpoints to the controlled preservation and release of molecular capacity.

## WET DORMANCY AS THROTTLED DYNAMICS IN *SACCHAROMYCES CEREVISIAE* SPORES

If *B. subtilis* endospores exemplify dormancy through enforced immobilization, *S. cerevisiae* spores illustrate a different strategy: dormancy achieved not by eliminating internal activity, but by throttling it (11). Although details remain to be worked out, I propose here that yeast dormancy is maintained by throttling dynamics within a hydrated, dynamic intracellular environment. In the dynamical-systems language introduced earlier, wet dormancy poses a distinct conceptual puzzle: how can local molecular clocks continue to tick while the global biological clock remains arrested? Yeast spores therefore shift the central question from “how are internal dynamics stopped?” to “how is ongoing microscopic activity prevented from propagating into global biological time?”

A defining physical feature of yeast spores is that they remain hydrated. Unlike *B. subtilis* endospores, yeast spores do not undergo massive cytoplasmic dehydration, nor do they accumulate Ca–dipicolinic acid or analogous agents that drastically reduce water abundance (5, 35–39). Instead, the intracellular environment of yeast spores behaves like a crowded, viscoelastic medium (40–43) in which molecular mobility and chemical reactions, including gene expression (11), are slowed but not abolished. Using fluorescence microscopy at single-spore resolution, a recent study directly demonstrated measurable protein mobility and gene expression dynamics in dormant yeast spores, establishing that yeast dormancy cannot be equated with physical arrest (11). Rather, yeast spores occupy a regime of low but detectable dynamics in which irreversible, ATP-dependent molecular processes persist even though developmental progression does not.

This hydrated, dynamic interior has important consequences for what remains possible during dormancy. Components can move, and macromolecular assemblies can reorganize. Critically, dormant yeast spores are neither transcriptionally nor translationally dead. The same recent study showed that dormant yeast spores incubated in PBS (i.e., without nutrients) at 30°C can both transcribe and translate, and that their gradual loss of viability is linked to the abundance of their stored RNA polymerases I–III gradually decreasing (11). Because nutrients are absent in PBS, any such transcriptional and translational activity must draw on stored molecular and energetic resources and therefore cannot be sustained indefinitely. In parallel, the spores’ capacity for gene expression decreases: both GFP reporter induction and endogenous transcription, visualized via incorporation of 5-ethynyl uridine, occurred in dormant spores in PBS but diminished as RNA polymerase levels fell over days (11). Moreover, this study showed that a decline in gene expression capacity in dormant yeast spores correlated with delayed awakening: yeast spores with severely reduced RNA polymerase content required substantially longer times—often many hours, with some requiring as much as ~12 h—to even start to germinate after abundant nutrients reappear (11). In these spores, detectable GFP production typically appeared only shortly before or concomitant with bud emergence. These observations place gene expression capacity not simply downstream of germination, but plausibly near a bottleneck controlling escape from dormancy: local irreversible events in transcription and translation can occur without nutrients, yet they do not automatically trigger the global transition to growth.

The behaviors of yeast spores mentioned above stand in sharp contrast to the logic of dry dormancy in *B. subtilis*. In *B. subtilis* spores, global time stops largely because local molecular clocks are arrested by dehydration and immobilization; transcription and translation are effectively impossible until rehydration and architectural remodeling occur. In dormant yeast spores, by contrast, the machinery for local processes is present and intermittently active (11), yet growth does not occur and life appears suspended at the global scale. The defining feature of wet dormancy is therefore not missing parts, but missing propagation: a failure of local activity to recruit the feedback loops that normally drive sustained metabolism, biosynthesis, and cell cycle progression.

How, then, is wet dormancy maintained? Consistent with the throttling framework introduced earlier, dormant yeast spores may constrain a small number of global control variables that cap system-wide throughput without eliminating underlying chemical processes. Throttling would not be a uniform slowing of all dynamics, but a hierarchy: some processes (e.g., diffusion, local rearrangements, intermittent transcription and translation) persist, while others (e.g., sustained translation programs, polarized growth, DNA replication, and cell wall remodeling) remain effectively blocked because one or more controlling variables stay below a threshold. In engine-like terms, components can still move, but the “power” required for global progression is capped.

Mechanistically, several candidate throttles are plausible and not mutually exclusive. Energy flux may be constrained: ATP is not eliminated, as in dry dormancy, but its availability or effective utilization could be limited in ways that prevent commitment to energetically expensive, self-amplifying programs. Cytosolic pH and ionic state, which are known to shift during entry into nongrowth states across biology, could modulate enzyme activity and macromolecular interactions without freezing the intracellular environment (42, 43). More generally, the coupling between gene expression and downstream growth-enabling and active-life processes may be below threshold: local activity occurs, but it fails to reliably propagate into sustained, global gene expression capable of driving coherent system-level dynamics associated with vegetative cell life. In state-space terms, the yeast spore explores a region in which local excursions occur, but robust access to the attractor corresponding to active life is rare.

A striking population-level consequence of throttling is heterogeneity in wake-up times. Even in isogenic populations exposed to uniform nutrients, yeast spores exhibit broad distributions of germination times, with some spores exhibiting very long delays of many hours. These timescales can exceed typical doubling times (11). Such behavior is difficult to reconcile with a biochemical program in which activity begins immediately upon nutrient addition and then accumulates gradually over time. In the aforementioned study that established ongoing, albeit sporadic, gene expression in dormant yeast spores without nutrients, gene expression reporters did not show slow, continuous buildup during extended delays. Instead, detectable GFP production appeared abruptly and typically only shortly before or concomitant with bud emergence (11). This pattern is more naturally explained by the presence of one or more bottlenecks—thresholded constraints that must be stochastically cleared before transcriptional and translational activity can propagate into global progression. In this view, nutrient addition reshapes the dynamical landscape to permit escape, but the timing of escape is set by rare internal configurations or multivariable threshold crossings rather than by uniformly slow kinetics. I note that heterogeneity in stored molecular resources, such as varying RNA polymerase content among spores, is one concrete mechanism by which spores can differ in their initial conditions within the throttled region of state space, and is therefore complementary to, rather than competing with, the throttling framework. Spores with lower stored resource levels would simply start further from the threshold, producing longer delays before crossing into global progression.

Seen this way, dormant yeast spores preserve nonequilibrium capacity not by freezing internal dynamics, but by maintaining a reorganizable intracellular environment that can, when multiple constraints are relieved, support sustained nonequilibrium activity (1). The benefit of this strategy is responsiveness; the cost is variability and unpredictability in awakening.

I note that wet dormancy, as defined here, is distinct from near-zero growth states such as those found in retentostat cultures (44, 45). In such states, local molecular processes remain coupled, at least in principle, to eventual global progression: cells grow, however, slowly. In wet dormancy, by contrast, global biological time is effectively arrested following sporulation, and the coupling between local processes and global progression is disrupted. The throttling framework predicts threshold-like transitions rather than gradual slowing, consistent with the abrupt appearance of GFP reporter expression observed in a recent study (11), rather than a gradual buildup. I acknowledge that the precise boundary between wet dormancy and very slow growth is an open question. This boundary is further informed by the recent discovery that the rate axis itself has a lower limit for coupled dynamics in yeast: vegetative cells cannot reduce cell cycle progression below a critical rate, as ROS accumulation competes with gene expression capacity; below this threshold, cells fail to complete the cell cycle and die (6). This suggests that a region of parameter space characterized by very low rates with intact coupling is not generally accessible to vegetative cells. From this perspective, the distinction between retentostat-like near-zero growth states (44, 45) and sporulation-derived dormancy is not solely one of rate but of dynamical organization: in retentostat cultures, local processes remain coupled to global progression, albeit at very low flux, whereas in wet dormancy, this coupling is effectively disrupted following sporulation. This represents a transition in dynamical organization rather than a simple extension of the slow-growth regime.

## COMPARING DRY AND WET DORMANCY AS DISTINCT REGIONS OF A PHYSICAL DESIGN SPACE

The preceding sections described two canonical forms of microbial dormancy. Because quantitative dynamical-systems explanations of dry and wet dormancy are not yet available, I use experimental observations and known molecular and physical constraints to propose a phenomenological organizing principle: dormancy strategies occupy distinct regions of a physical design space defined by hydration, molecular mobility, energetic flux, and the coupling between local molecular clocks and the global biological clock.

In this framing, dry and wet dormancy are not merely qualitative labels but endpoints along shared physical axes—axes of hydration, molecular mobility, energetic flux, and clock coupling—rather than points on a growth-rate continuum. Dry dormancy, exemplified by *B. subtilis* endospores, is characterized by an intracellular environment engineered into a low–water-activity, low-mobility regime in which molecular diffusion and many reaction fluxes are strongly suppressed. Consistent with this physical state, I interpret dry dormancy as a regime in which global biological time is arrested largely because local molecular clocks are rendered slow and/or nonproductive. In this view, nonequilibrium capacity (NEC) is stored in a physically protected, immobilized configuration, and exit from dormancy appears to proceed through coordinated physical reversal, such as rehydration, restoration of mobility, and reactivation of core molecular machinery.

Wet dormancy, exemplified by *S. cerevisiae* spores, occupies a qualitatively different region of this design space. The intracellular environment remains hydrated and dynamic; local molecular clocks continue to tick; and irreversible processes such as transcription and translation can occur, albeit extremely slowly. Yet global biological time remains arrested. I hypothesize that this occurs because ongoing microscopic activity is throttled below one or more thresholds required for self-amplifying feedback and sustained systems-level progression characteristic of vegetative life (local processes persist but fail to propagate into global progression). In this hypothesis, NEC is preserved not by freezing dynamics but by maintaining a reorganizable intracellular environment while constraining the couplings that would otherwise drive growth and other forms of vegetative life.

Table 1 summarizes these distinctions by placing *B. subtilis* and yeast spores along shared physical and dynamical axes, highlighting immobilization and throttling as alternative strategies for suspending global time, and illustrating that dry and wet dormancy define useful endpoints rather than exhaustive categories. Intermediate strategies—such as bacterial persisters (46–50), viable-but-nonculturable bacteria (51–53), or quiescent eukaryotic cells (35, 54–56)—likely occupy regions between these extremes, combining partial immobilization with partial throttling. The value of the dry–wet distinction is therefore not complete classification but clarification of the axes along which dormancy strategies vary.

**TABLE 1:** Comparison of dry-dormancy strategy in *B. subtilis* spores and wet-dormancy strategy in *S. cerevisiae* spores

Feature	*Bacillus subtilis* (dry dormancy)	*Saccharomyces cerevisiae* (wet dormancy)
Intracellular hydration	Strongly reduced; low water activity	High; cytoplasm remains hydrated
Cytoplasmic physical state	Low-mobility, glass-like, highly constrained	Viscoelastic, crowded, reorganizable
Molecular diffusion	Extremely suppressed in dormancy	Slowed but measurable
DNA organization	SASP-bound, altered conformation	Transcription-permissive on reawakening; dormancy-specific chromatin organization incompletely resolved
Transcriptional activity	Effectively impossible until rehydration	Can occur prior to germination
Translation machinery	Preserved/poised; specific mechanisms incompletely established	Preserved; reactivation upon nutrient addition
Energetic flux	Near-zero; ATP largely unavailable	Low but nonzero; throttled
Primary dormancy enforcement	Physical immobilization and dehydration	Proposed throttles (energetic/physicochemical thresholds; e.g., ATP flux, pH/ionic state)
NEC storage strategy	Stored in immobilized, protected macromolecular configurations (glass-like core; protected DNA/ribosomes)	Stored in a hydrated, reorganizable cytoplasm; NEC expression capped by throttles (energy/physicochemical state)
Coupling between local and global clocks	Local clocks largely suppressed; coupling to global progression effectively off	Local clocks active; coupling to global progression remains below threshold

Situating other states in this design space is instructive. Desiccated nonspore-forming bacteria, including organisms that survive in anhydrobiotic conditions, likely occupy a region near the dry end of the hydration axis: low water activity and suppressed molecular mobility, even though they arrive at this state without a sporulation program (57). Anhydrobiotic organisms more broadly share these physical features with dry dormancy. Other bacterial spore-formers beyond *B. subtilis*, such as *Streptomyces*, produce exospores through distinct developmental programs and may occupy related but not identical regions of the design space. Deep-sea microbes with potentially millennial metabolic turnover may occupy an intermediate position, with some residual local activity but very slow global progression, perhaps closer to the wet end of the throttling axis (58). These placements are necessarily provisional, as the quantitative physical and molecular data needed to characterize these states along the proposed axes are not yet available.

## CONCLUSION: SUSPENDING LIFE BY IMMOBILIZATION AND THROTTLING

Dormancy has long been described in biological terms, such as sporulation programs, protective layers, or metabolic shutdown. But these descriptions alone do not completely explain how life can be paused and reliably restarted (1, 30). Here, I argued that microbial spores offer a unique opportunity to reframe dormancy as a problem in dynamical systems and physical state space. By contrasting *B. subtilis* endospores and *S. cerevisiae* ascospores, I proposed two distinct strategies for suspending life: enforced immobilization and throttling of dynamics.

In dry dormancy, dehydration and mechanical constraint push the system into a low-mobility regime in which productive chemistry is kinetically inaccessible; global biological time is arrested largely because local molecular clocks themselves are suppressed. Awakening requires a coordinated physical reversal that restores mobility and recouples local processes to global progression. In wet dormancy, local clocks continue to tick while global biological time remains arrested; I propose that this is achieved by throttling, in which ongoing microscopic activity is constrained below thresholds required for self-amplifying feedback and sustained systems-level progression. Awakening becomes a threshold-crossing problem, naturally producing heterogeneity and long delays even in genetically identical populations.

At present, this dynamical-systems framework remains phenomenological. A quantitative dynamical-systems treatment of dry and wet dormancy—explicitly modeling state variables, thresholds, and coupling strengths—has yet to be developed. Nevertheless, the experimental observations reviewed here strongly suggest that suspending life does not require eliminating all dynamics. Instead, life can be paused either by suppressing motion itself or by allowing motion to continue while preventing it from becoming productive.

By making this distinction explicit, I aim to reframe dormancy in dynamical terms: as the control of how global biological time is stopped, stored, and restarted, either by suppressing local molecular clocks or by allowing them to run while decoupling them from global progression. Such a perspective is relevant not only for spores but more broadly for developing a physical theory of living matter that spans growth, dormancy, and revival.
